# Kinetic Study of the Maillard Reaction in Thin Film Generated by Microdroplets Deposition

**DOI:** 10.3390/molecules27185747

**Published:** 2022-09-06

**Authors:** Chiara Salvitti, Giulia de Petris, Anna Troiani, Marta Managò, Andreina Ricci, Federico Pepi

**Affiliations:** 1 Department of Chemistry and Drug Technologies, Sapienza University of Rome, P.le Aldo Moro 5, 00185 Rome, Italy; 2 Department of Mathematics and Physics, University of Campania, L. Vanvitelli, Viale Lincoln 5, 81100 Caserta, Italy

**Keywords:** Maillard reaction, microdroplets, thin film reactions, ESI mass spectrometry

## Abstract

The Maillard reaction kinetics in the confined volume of the thin film produced by ESI microdroplet deposition was studied by mass spectrometry. The almost exclusive formation of the Amadori product from the reaction of D-xylose and D-glucose toward L-glycine and L-lysine was demonstrated. The thin film Maillard reaction occurred at a mild synthetic condition under which the same process in solution was not observed. The comparison of the thin film kinetics with that of the reaction performed in solution showed strong thin film rate acceleration factors.

## 1. Introduction

The electrospray ionization (ESI) source of a mass spectrometer operating under ambient conditions can be considered as a miniaturized laboratory by which the reactions taking place at the liquid–gas interface can be easily studied [1,2,3,4,5,6,7]. The charged microdroplets generated in the ESI process represent confined reaction volumes where the rate of the chemical reactions can be accelerated hundreds of times with respect to the same processes performed in bulk [8,9,10,11,12,13,14,15]. The reaction acceleration was attributed to the greater mobility of reagents at the interface, to the extremely high pH values, and to the strong electric field created at the droplets’ interface [16,17,18,19].

**Scheme 1:**
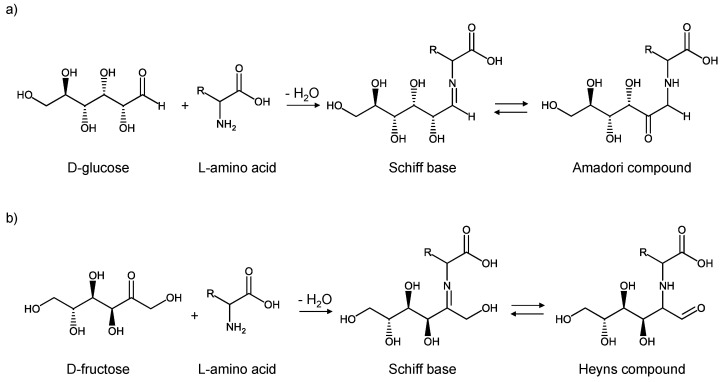
The first step of the Maillard reaction: (**a**) the reaction involving D-glucose and (**b**) the reaction involving D-fructose.

The continuous landing of the electrosprayed microdroplets onto a target solid surface allows for the formation of a stable thin film that is characterized by an extremely high reagent concentration. Under these conditions, the final products of the reactions can be collected by continuously supplying the microdroplets onto the target surface. Based on the results obtained on the gas-phase ionic reactions of biomass-derived sugars [20,21,22,23], we have recently extended their gas-phase reactivity to the confined volume of the thin films as a further step to tentatively scale up the observed processes for industrial applications [10]. In the present study, the thin film formation by microdroplet deposition was exploited to investigate another fundamental process involving sugars, the Maillard reaction, a milestone reaction of organic chemistry between reducing sugars, and the free amino group of amino acids [24,25]. The Maillard reaction is the primary process responsible for the formation of desirable or undesirable flavored and colored products in cooked foods [26,27,28,29,30] and occurs in vivo in mammalian organisms where glycosylation is one of the most common protein post-translational modification processes [31,32,33,34]. The initial stage of the reaction involves the addition of the free amino group of amino acids (in particular, the L-lysine ε-amino groups of proteins but also the α-amino groups of terminal amino acids) to the sugar carbonyl, resulting in the reversible formation of a Schiff base from the removal of a water molecule. The Schiff base formed rearranges into Amadori or Heyns products depending on the nature of the sugar involved [35,36,37]. Aldoses such as D-glucose lead to the Amadori compound whereas ketoses such as D-fructose give rise to the Heyns intermediate (Scheme 1).

In food heating treatment, the reaction continues until the formation of volatile compounds that are usually associated with food fragrance and aroma. From this point of view, the final Maillard reaction products can be exploited to produce synthetic flavoring substances that can be added to processed foods [38,39,40]. However, such volatile compounds are easily lost from foods, thus attenuating their flavor contribution. The use of Amadori or Heyns intermediates, instead of their volatile decomposition products, is a promising method for controlled food flavor enhancement. There are several studies on the preparation of Maillard reaction intermediates in the aqueous phase using amino acids and sugars, however, due to the easy degradation of the initial Schiff base or Amadori and Heyns products, the whole process remains quite complex [38,41]. 

Recently, Cooks and co-workers studied the accelerated Schiff base formation from the reaction between primary amines and saccharides for saccharide discrimination in tissues and single cells in a thin film reactor where concentrated solution droplets had been left to dry over time [42]. The main objective of the present work is the study of the Maillard reaction in the thin film environment as a potential method to synthesize flavoring precursor products as well as to investigate the kinetics of the process by developing a procedure where the thin film containing a fixed amount of the reagents is continuously formed by pure solvent microdroplet deposition. 

## 2. Results and Discussion

To test the Maillard reaction in the thin film, the reactivity of aldose and ketose sugars (D-glucose, D-xylose, and D-fructose) toward L-glycine, the simplest amino acid, and L-lysine, carrying an additional ε-amino group, was probed by collecting the microdroplets generated by the Z-spray ESI source of a Q-TOF mass spectrometer onto a stainless-steel surface. The starting solutions were prepared by mixing an equimolar quantity of the sugar and the amino acid in a 3:1 H_2_O/CH_3_OH solution. The ESI mass spectra of the starting solutions reflecting the ionic composition of the microdroplets are reported in the Supplementary Materials (Figures S1 and S2). The spectra of the starting L-glycine/sugar solutions were dominated by the [M]Na^+^ and [M_2_]Na^+^ adducts characteristic of the starting sugar bound to the ubiquitous sodium cations. The lack of any ionic signal from L-glycine can be attributed to the presence in the solution of its zwitterion. 

In contrast, the easy protonation of the L-lysine ε-amino group under ESI conditions leads to the almost exclusive observation in the spectra of the L-lysine/sugar mixtures of the [Lys]H^+^ and [Lys-NH_3_]H^+^ ionic species at *m*/*z* 147 and *m*/*z* 130, respectively. No ionic species attributable to Maillard reaction products were observed, thus demonstrating that no reaction occurs in the microdroplet beam prior to their deposition onto the solid surface. 

**Figure 1:**
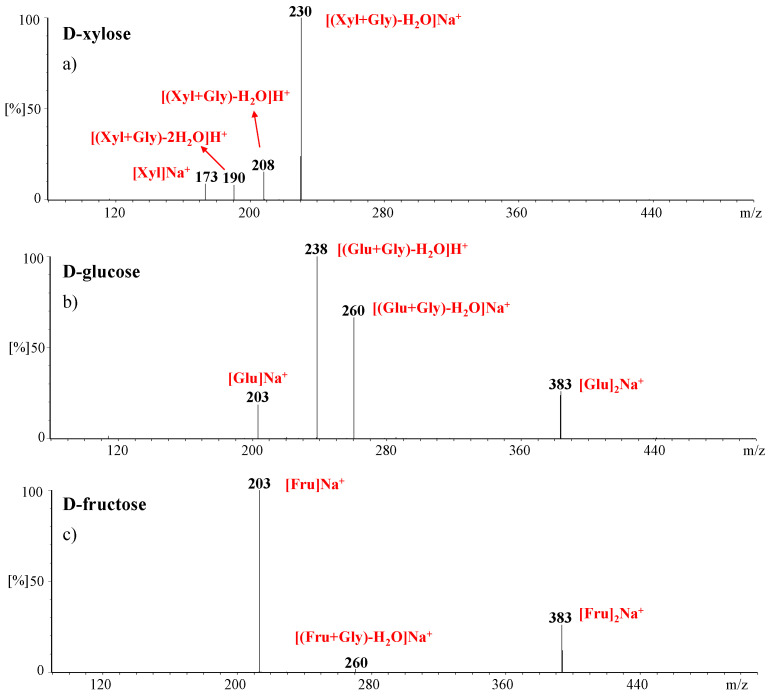
The ESI mass spectra of the solutions obtained by rinsing the solid precipitate generated by the ESI microdroplet deposition of: (**a**) 1 mL of 1:1 L-glycine/D-xylose 10^−3^ M H_2_O/CH_3_OH 3:1 solution; (**b**) 1 mL of 1:1 L-glycine/D-glucose 10^−3^ M H_2_O/CH_3_OH 3:1 solution; (**c**) 1 mL of 1:1 L-glycine/D-fructose 10^−3^ M H_2_O/CH_3_OH 3:1 solution.

**Figure 2:**
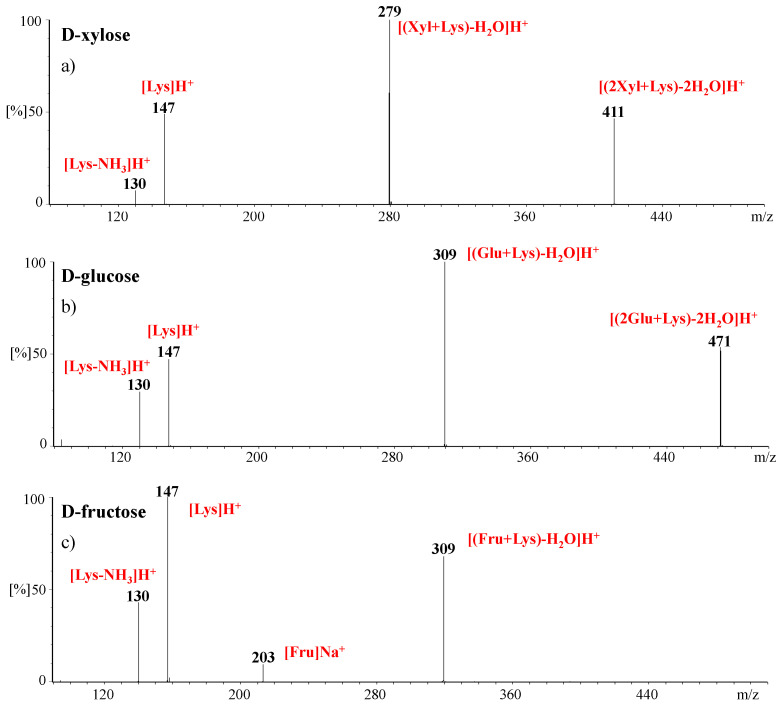
The ESI mass spectra of the solutions obtained by rinsing the solid precipitate generated by the ESI microdroplet deposition of: (**a**) 1 mL of 1:1 L-lysine/D-xylose 10^−3^ M H_2_O/CH_3_OH 3:1 solution; (**b**) 1 mL of 1:1 L-lysine/D-glucose 10^−3^ M H_2_O/CH_3_OH 3:1 solution; (**c**) 1 mL of 1:1 L-lysine/D-fructose 10^−3^ M H_2_O/CH_3_OH 3:1 solution.

The deposition of the ESI microdroplets onto the stainless-steel surface generates a thin film from which a brown precipitate separates during the reaction time (typically 50 min). The ESI mass spectra of the rinsed precipitates produced by the different amino acid/sugar thin film systems are reported in Figure 1 and Figure 2, respectively. 

In the L-glycine/D-xylose thin film (Figure 1a), the extensive formation of the Maillard product, corresponding to the addition of the amino acid to the sugar with the concomitant removal of a water molecule, is evidenced by the protonated and sodiated ions at *m*/*z* 208 and *m*/*z* 230, respectively. An ionic species corresponding to the loss of an additional water molecule can also be observed at *m*/*z* 190.

In the L-glycine/D-glucose system analogous abundant protonated and sodiated ions at *m*/*z* 238 and *m*/*z* 260, corresponding to the first step of the Maillard reaction, were also present (Figure 1b). Conversely, only traces of the sodiated Maillard product at *m*/*z* 260 were observed with D-fructose (Figure 1c).

Passing to the L-lysine/D-xylose and L-lysine/D-glucose solutions, the Maillard products are evidenced by the formation of the protonated species at *m*/*z* 279 and *m*/*z* 309, respectively (Figure 2a,b).

In these systems, it is also evident that the addition of a second sugar molecule that leads to the ionic products at *m*/*z* 411 and *m*/*z* 471 is due to the presence of two amino groups in the L-lysine structure. It is interesting to underline that all of the above-mentioned ionic species, proving the occurred Maillard reaction, represent the most abundant ions of the ESI spectra of the aldose containing systems. Moderate formation of the Maillard product was observed when L-lysine was allowed to react with D-fructose, which, moreover, was limited to the addition of a single sugar molecule (Figure 2c). Considering the lack of D-fructose addition product with L-glycine, it is reasonable to assume that its addition to L-lysine occurs in the more reactive ε NH_2_ group, thus confirming the lower reactivity of the amino acids α amino group with ketoses. In particular, the scarce formation of the early stage Maillard reaction products in D-fructose/amino acid systems was attributed to the formation of advanced Maillard products and/or to its easy degradation through the caramelization reaction [43,44,45]. Nevertheless, in the thin film systems containing D-fructose, the existence of these processes can be excluded as demonstrated by the almost unaltered ionic signals of the starting reactants observed in the mass spectra of the rinsed precipitates (Figure 1c and Figure 2c).

**Scheme 2:**
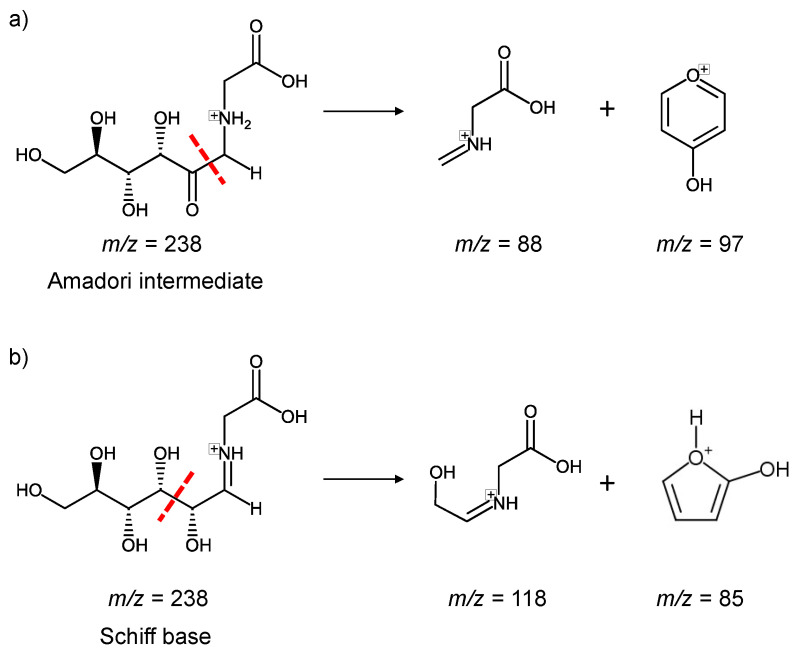
The main diagnostic fragmentations of: (**a**) Amadori intermediate and (**b**) Schiff base.

The results of a recent mass spectrometric study by Xing et al. [46] about the fragmentation pattern of the L-glycine/D-glucose adduct allows them to distinguish whether the amino acid/sugar addition products were characterized by the structure of the initially formed Schiff base or by the structure of its Amadori rearrangement isomer. The main diagnostic MS/MS fragmentations differentiating the Schiff base from the Amadori intermediate are reported in Scheme 2.

**Figure 3:**
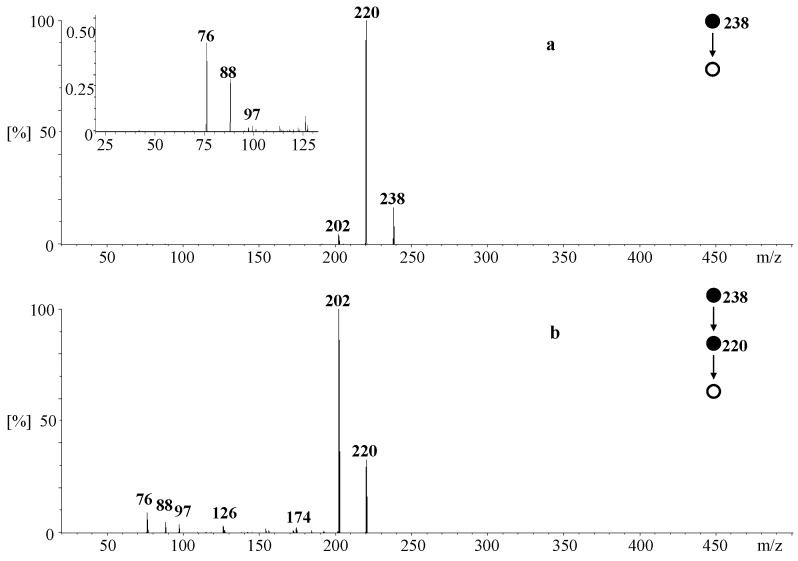
The ion trap MS^2^ (**a**) and MS^3^ (**b**) CID spectra of the L-glycine/D-glucose condensation product at *m*/*z* 238. The inset shows the diagnostic fragmentation peaks in the *m*/*z* 25–130 interval of MS^2^.

The ions at *m*/*z* 88 and *m*/*z* 97, deriving from the cleavage of the monosaccharide carbon chain at the C1–C2 position, were taken as diagnostic peaks for the occurrence of the Amadori product, whereas the ions at *m*/*z* 118 and *m*/*z* 85 were considered indicative of the formation of the Schiff base. Taking into account, these results, the L-glycine/D-glucose condensation product at *m*/*z* 238 was mass-isolated and subjected to MS^n^ experiments into an ion-trap mass spectrometer (Figure 3). 

The protonated species at *m*/*z* 238 predominantly dissociate by releasing two water molecules, leading to the formation of the fragment ions at *m*/*z* 220 and *m*/*z* 202, respectively (Figure 3a). The dehydration products predictably arise from the -OH groups of the monosaccharide moiety, as also demonstrated by our previous studies on the gas-phase decomposition of hexose and pentose sugars [20,21,22,23]. However, despite being highly represented in the CID mass spectrum, these ionic species are not useful in discriminating between the above-mentioned isobaric species. In contrast, the minor fragments ion at *m*/*z* 88 and *m*/*z* 97 account for the formation of the Amadori intermediate at the expense of the Schiff base (Figure 3a, inset). The same product ions are also present in the CID mass spectrum of the dehydration product at *m*/*z* 220 obtained from the precursor ion at *m*/*z* 238 through a second cycle of isolation/fragmentation (Figure 3b). The lack of any Schiff base diagnostic fragments clearly indicates the predominant formation of the Amadori rearrangement product from the Maillard reaction between L-glycine and D-glucose performed in the thin film. Similar experiments were also performed on the sodiated L-glycine/D-glucose product at *m*/*z* 260 with no appreciable evidence for the isomeric discrimination. Analogous results were obtained for the corresponding L-glycine/D-xylose product ions at *m*/*z* 208, also showing the diagnostic fragment at *m*/*z* 88, whereas, in the case of all the L-lysine/sugar systems, the breakage of the amino acid scaffold predominates over the cleavage of the monosaccharide carbon chain and no diagnostic fragments for correct ion attribution were detected. However, it is reasonable to suppose that also in the L-lysine systems, the Schiff base evolves to the formation of the Amadori intermediate with D-glucose and D-xylose aldose sugars.

### 2.1. The Effects of The ESI Z-Spray Desolvation Temperature

**Figure 4:**
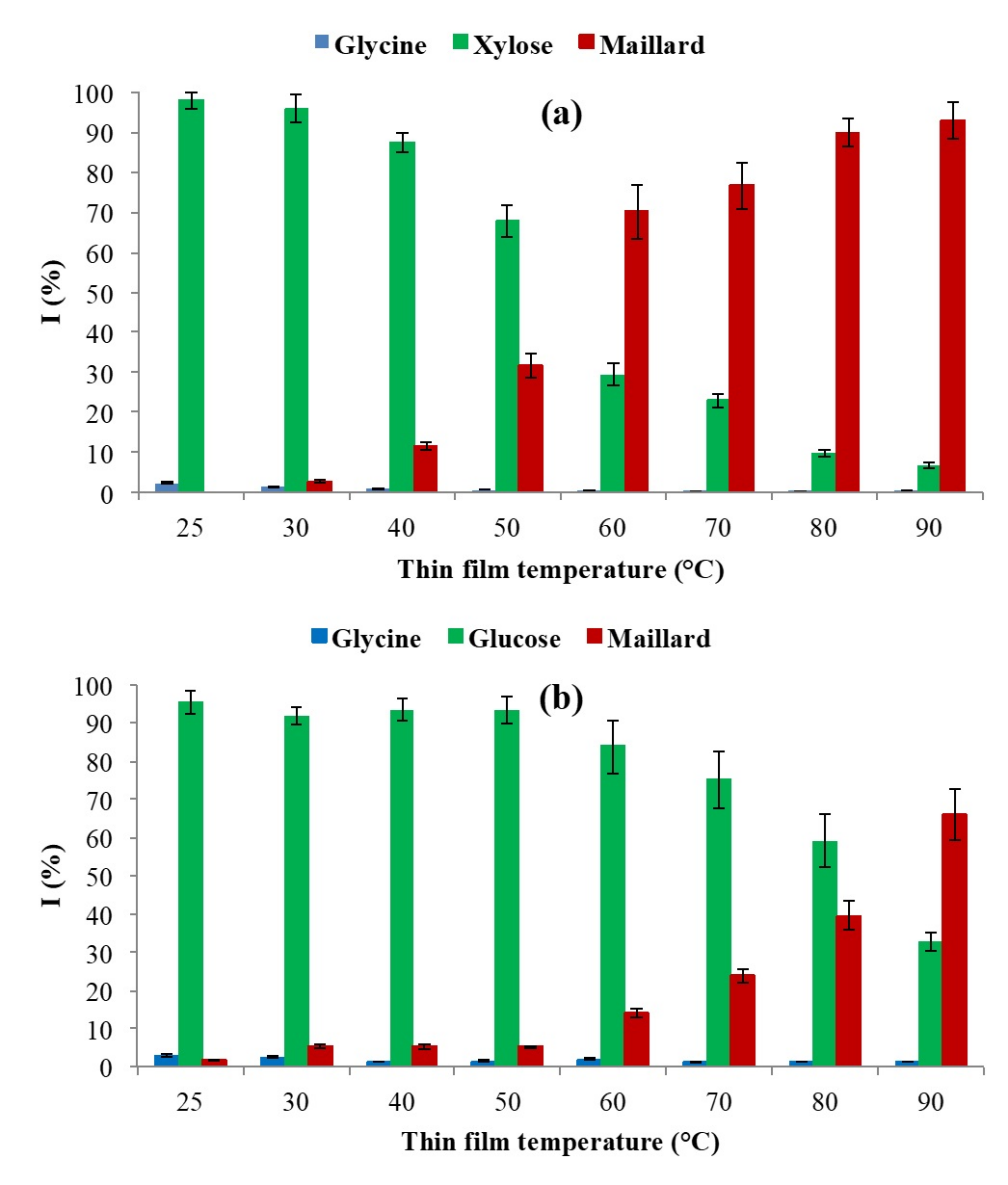
The reactants and Maillard reaction products (%) versus thin film temperatures (°C): (**a**) L-glycine/D-xylose mixtures and (**b**) L-glycine/D-glucose mixtures.

**Figure 5:**
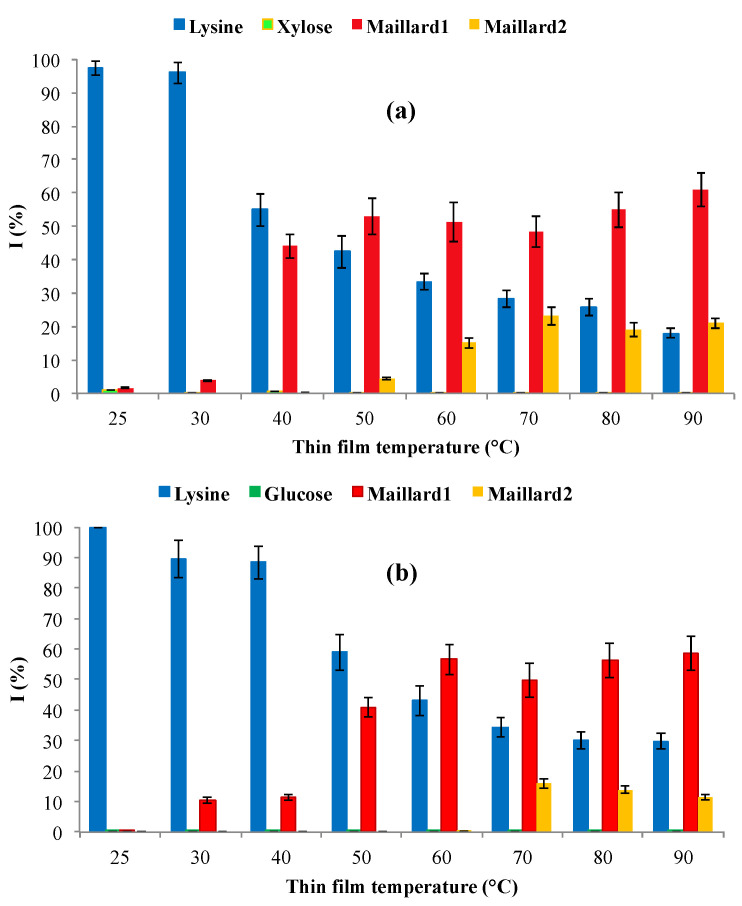
The reactants and Maillard reaction products (%) versus thin film temperatures °C. (**a**) L-lysine/D-xylose mixtures and (**b**) L-lysine/D-glucose mixtures.

Since the Maillard reaction is a thermal process mainly affected by the reaction temperature, the effect of the ESI source desolvation gas temperature, promoting solvent evaporation in the deposited thin film, was deeply investigated. The temperatures of the desolvation nozzle were varied from 50 to 350 °C and the corresponding actual temperatures of the collected thin film measured by a thermocouple. Commonly, the actual temperatures of the solid surface were around 30% of the set desolvation gas temperature (i.e., a desolvation gas temperature of 200 °C corresponded to a thin film temperature of about 60 °C). In Figure 4 and Figure 5, the sum of the Maillard products’ relative intensities with respect to the unreacted sugar and amino acid was plotted as a function of the actual thin film temperatures. The values reported represent the average of three different measurements. Considering the low reactivity of D-fructose, these experiments were limited to D-xylose and D-glucose.

As underlined in the previous paragraph, L-glycine is scarcely ionized under the ESI conditions. In the reaction mixtures containing this amino acid, the abundances of the unreacted species were derived by the decrease in the sugar sodiated ions. Conversely, L-lysine is efficiently ionized by protonation onto its basic ε amino group, thus the reactant consumption is measured by the decrease in this very intense ionic species. This discrepancy does not allow, at this stage, for a comparison of the reactivity of the two amino acids, whereas it is possible to draw some considerations regarding the different reactivity of the two sugars with respect to the same amino acid. In the L-glycine/D-xylose mixture (Figure 4a), the Maillard product (red bars) predominates over the unreacted species (green bars) at a temperature of at least 60 °C and represent almost 90% of the whole ionic current at a temperature of 80 °C.

A temperature of about 90 °C is necessary to obtain a Maillard product ion intensity of 70% in the reaction involving D-glucose (Figure 4b). It seems to be evident that D-xylose is more reactive toward L-glycine than D-glucose. Passing to the L-lysine systems, the two addition products were labeled as Maillard 1, assuming that the first sugar molecule is bound to the ε NH_2_ group, and Maillard 2, with an additional sugar molecule, is bound to the α NH_2_ moiety. Higher temperatures with respect to those observed for D-xylose catalyze the addition reaction of D-glucose to L-lysine. 

The Maillard 1 reaction products reached a maximum even at 50 °C in the case of D-xylose (Figure 5a), whereas a similar product distribution in the D-glucose solution can be achieved around 60 °C (Figure 5b). The addition of the second sugar molecule started at a temperature of around 50 °C in the case of D-xylose and at least 70 °C for D-glucose.

### 2.2. Maillard Reaction Kinetics in Thin Film with Respect to The Bulk Solution

**Figure 6:**
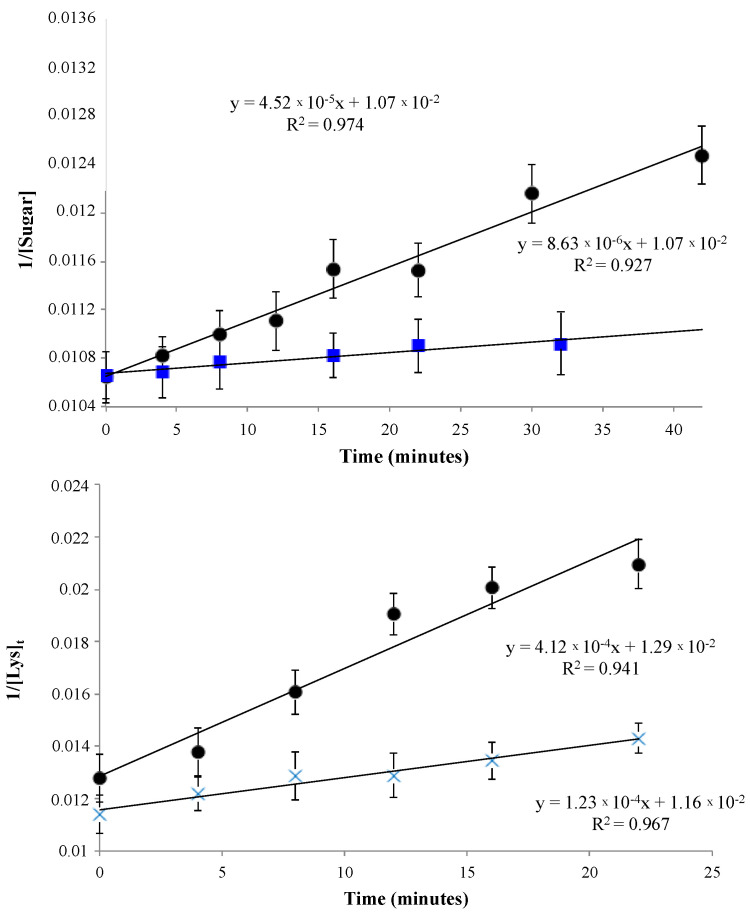
The kinetic plots of the second-order thin film Maillard reaction for the (**a**) L-glycine/sugar systems and (**b**) L-lysine/sugar systems.

The kinetics of the observed first step of the Maillard reaction in the thin film was studied by extending over time the life of the thin film containing a fixed amount of the two reactant molecules. To this end, the pure solvent was infused for different periods of time, having previously deposited the 1:1 10^−3^ M amino acid/sugar mixture for 5 min onto the collecting surface. In this way, the thin film was continuously renewed without adding a new amount of the reagents and ensuring a constant reaction volume. By using this procedure, it is not possible to estimate an absolute value of the reaction kinetic constant since the actual thin film volume and reactant concentration cannot be known. Nevertheless, relative reaction rates can be obtained by the comparison of the kinetic data measured in the different sugar/amino acid systems in a self-consistent kinetic model [42]. The bimolecular Maillard reaction was considered as a second-order reaction as has usually been reported in the literature, even if some kinetics studies claim that this process is a zero- and first-order reaction under particular synthetic conditions [47,48]. We assumed that the concentration C of a reactant species can be described by its ion abundance and considering the value of 1/C was plotted against the reaction time obtaining the best-fit lines reported in Figure 6. The slopes of the best-fit lines were hence used to measure the ratio between the kinetics constants of the different sugar/amino acid systems, assuming that:

(1) $$\frac{k_{\mathrm{system}1}}{k_{\mathrm{system}2}}=\frac{{\mathrm{slope}}_{\mathrm{system}1}}{{\mathrm{slope}}_{\mathrm{system}2}}$$

As previously described, due to the different amino acid ionization efficiencies, the best fit lines of the D-xylose and D-glucose reactions toward L-glycine were constructed by following the sugar ions’ relative intensity, whereas, in the reactions involving L-lysine, the plot was based on the L-lysine ions relative intensity decrement. Since the kinetics data of the different mixtures have to be compared under the same experimental conditions, and in the case of L-lysine, it is necessary to avoid the presence of the competing addition of the second sugar molecule, the kinetics of all reactions was studied at a thin film temperature of 40 °C (desolvation gas temperature of 100 °C). Moreover, this temperature represents the higher temperature at which the extensive reactant conversion into the products during the initial deposition is prevented.

The slopes of the lines relevant to the reactions involving D-xylose were clearly higher than those found in the D-glucose systems. In particular, compared to the reaction involving D-glucose, the reactions of D-xylose with L-glycine and L-lysine were about five and three times faster, respectively. These results are consistent with the well-recognized different sugars’ reactivity toward amino acids observed in solution that, generally, showed a faster Maillard pentose sugar reaction than hexose [49].

Although the ionic species used to construct the kinetics plot for the two amino acid solutions were different, from the relative slopes obtained, it can reasonably be stated that L-lysine is more reactive than L-glycine by a factor of about 10 with both sugars tested. This acceleration factor underlines the greater reactivity of the amino acids ε amino group with respect to the α.

To establish the rate acceleration factors (RAF) [50] of the Maillard reaction performed by the microdroplet thin film deposition process with respect to the same reaction in bulk, 10 mL of the 3:1 H_2_O/CH_3_OH 10^−3^ M amino acid/sugar 1:1 solutions were heated under reflux for 1 h. No products were detected in bulk at 40 °C. To measure a lower limit of the RAF, the bulk reactions were hence tested using 10^−1^ M solutions of the reactants and the temperature raised to 100 °C. In addition, by using stronger synthetic conditions, no reaction products were observed for the L-glycine/D-glucose system. Conversely, small amounts of Maillard products were observed for the reactions of D-xylose with L-glycine and L-lysine and D-glucose with L-lysine. Aliquots of the reaction mixtures were sampled at different reaction times and the kinetics of the reactions were followed by analyzing the product concentration under the same mass spectrometric ESI conditions used to study the thin film reaction kinetics (Figure S3 Supplementary Materials). The whole slopes measured from the kinetic plots of the thin film and bulk reactions with the derived RAF are reported in Table 1.

Taking into account that the bulk reactions were conducted at a higher solution temperature than that used for the thin film (100 °C versus 40 °C), it is evident that the strong acceleration of the first dehydration step of the Maillard reaction in the thin film environment.

The reactions of D-xylose toward L-glycine and L-lysine were accelerated by factors ranging from 14 to 22. Moreover, it is interesting to underline that D-glucose, which was less reactive than D-xylose toward the amino acids used both in solution and in the thin film, showed a high thin film RAF that, in the case of L-lysine, was at least double with respect to that observed with D-xylose (45.4).

## 3. Materials and Methods

### 3.1. Reagents

D-xylose, D-glucose, D-fructose, L-glycine, L-lysine, solvents, and all other chemicals were purchased from Sigma-Aldrich Ltd. (St. Louis, MO, USA) and used without further purification.

### 3.2. Mass Spectrometric Experiments

Monosaccharide (D-xylose, D-glucose, or D-fructose) and amino acids (L-glycine and L-lysine) were mixed in a 1:1 ratio and dissolved in H_2_O/MeOH (3:1 *V/V*) at millimolar concentration. The starting solutions thus obtained were infused into the Z-spray source of a quadrupole-time-of-flight Q-TOF Ultima mass spectrometer (Micromass, Manchester, UK), previously adapted to microdroplet reaction studies [51] and operating in positive ion mode. Typical source potentials were as follows: capillary 4 kV, cone 60 V, RF lens-1 70 V. Nitrogen was used as a desolvation gas at a flow rate of 100 L h^−1^, source and desolvation temperatures were set at 80 and 250 °C, respectively.

After acquiring a zero-time mass spectrum in the 50–500 *m*/*z* range, 1 mL of the starting solution was electrosprayed at a syringe pump flow of 20 µL min^−1^ through the ESI source and the charged microdroplets generated at the capillary tip were deposited onto a stainless-steel plate held 3.0 cm away from the capillary exit. Hence, a total reaction time of 50 min can be estimated by considering the sprayed solution volume and the syringe flow rate.

The precipitate obtained by microdroplet deposition was weighed to estimate the recovery yield and collected into a vial by rinsing the stainless-steel plate with 1 mL of the same solvent mixture used to prepare the starting solution. As an example, to give an estimation of the scale of the reaction, in a standard experiment, 0.230 mg of D-xylose was left to react with 0.280 mg of L-lysine or 0.120 mg of L-glycine. Similar quantities were used for D-glucose. Typical recovery yields were around 90% of the solid starting materials. The dissolved precipitate was mass-analyzed under the ESI experimental conditions previously employed to acquire the zero-time mass spectrum. The unreacted reagents and final product abundances were derived from their ion intensities without applying any correction due to the different ionization efficiencies. Changes in the product ion intensities were then studied by varying the ESI spray desolvation temperature (50–350 °C). The reaction products were in-depth structurally characterized by tandem collision-induced dissociation (CID) experiments. Multiple stages of fragmentations were obtained by using an AmaZon SL ion trap instrument operating in the positive ion mode (Bruker, Bremen, Germany). MS*^n^* experiments were performed by isolating the reactant ions with a width of 1 *m*/*z* and subjecting them to dissociation. 

Kinetic experiments were performed by depositing the starting solution for 5 min at 40 °C and subsequently spraying the H_2_O/CH_3_OH mixture, without reagents, onto the thus formed precipitate for a time ranging between 0 and 45 min. In this manner, the reagent concentration and thin film thickness were kept constant over time. Five minutes was the shortest time to deposit a sufficient amount of reactants that could be converted in detectable products by MS, and 40 °C was the highest temperature that avoided the reactant conversion into the products during the initial deposition. 

The rate constant k was estimated according to the integrated second-order rate equation:

(2) $$\frac{1}{C}=\mathrm{kt}+\frac{1}{C_{0}}$$

where C and C_0_ are the concentration of the reactant at times t_0_ and t (i.e., the relative ion abundance of the reactant in the registered mass spectra).

All of the reactions were also performed in bulk by heating 10 mL of 10^−3^ and 10^−1^ M solutions of monosaccharide/amino acid for one hour under reflux in a 50 mL reaction vessel. During this time, aliquots of the reaction mixture were sampled at different reaction times, quenched, cooled, and diluted to millimolar concentration for mass spectrometric analysis. Based on the bulk results, the rate acceleration factor (RAF) was calculated for each reaction by comparing the kinetic parameter measured for the thin-film system with the corresponding value measured for the reaction in solution [47]. 

## 4. Conclusions

The Maillard reaction is one of the milestone processes of food chemistry, responsible for the flavor and nutritional value of food products. In the last years, the Maillard reaction has demonstrated great potential in the preparation of processed food flavorings, which are required to formulate more acceptable products for consumers. Here, we demonstrated the possibility of synthesizing an almost pure Amadori compound, taking advantage of the accelerated reactivity in the confined volume of thin films formed by ESI microdroplet deposition. The kinetic studies of the thin-film reactions between D-xylose and D-glucose with L-glycine and L-lysine show strong acceleration factors with respect to the same reactions in bulk. L-glycine glycosylation occurs in the thin-film environment by using mild synthetic conditions under which the same reaction in solution is not observed. This small volume procedure may represent a useful synthetic method to obtain a Maillard reaction product that, almost in principle, can be scaled up for industrial applications. 

## Figures and Tables

**Table 1:** The kinetic parameters and derived RAF for the investigated thin film and bulk reactions.

System	Slope (Thin Film)	Slope (Bulk)	RAF
D-xylose/L-glycine	4.52 × 10^−5^	3.20 × 10^−6^	14.1
D-xylose/L-lysine	4.12 × 10^−4^	1.88 × 10^−5^	21.9
D-glucose/L-glycine	8.63 × 10^−6^	-	∞
D-glucose/L-lysine	1.23 × 10^−4^	2.71 × 10^−6^	45.4

## Data Availability

Not applicable.

## Supplementary Materials

The following supporting information can be downloaded at: https://www.mdpi.com/article/10.3390/molecules27185747/s1, Figure S1: The ESI mass spectra of the starting L-glycine/sugar solutions; Figure S2: The ESI mass spectra of the starting L-lysine/sugar solutions; Figure S3: The kinetic plots of the reactions performed in bulk.

## Sample Availability

Samples of the compounds are not available from the authors.
