# Supplementary material for: Kinetic Study of the Maillard Reaction in Thin Film Generated by Microdroplets Deposition
Source: Molecules. 2022 Sep 6;27(18):5747. doi: 10.3390/molecules27185747 (PMC9504576; doi:10.3390/molecules27185747)
Supplement: Supplementary file 1 [file molecules-27-05747-s001.zip › molecules-1842938-supplementary.pdf]

Kinetic Study of The Maillard Reaction in Thin Film Generated by Microdroplets Deposition

Chiara Salvitti*, Giulia de Petris, Anna Troiani, Marta Managò, Andreina Ricci and Federico Pepi[a]*

Supplementary Materials

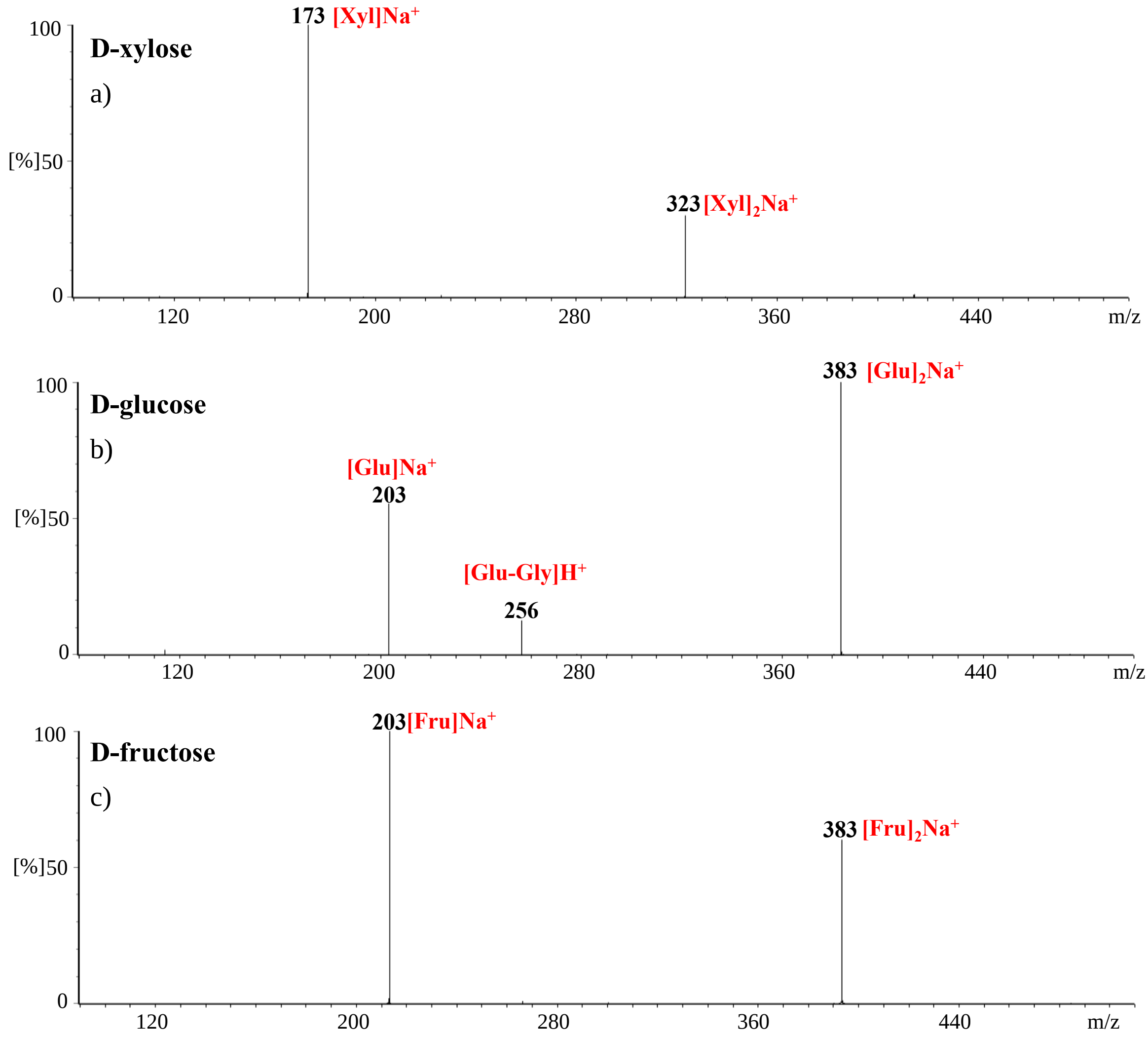

Figure S1: The ESI mass spectra of the starting solutions: a) 1 mL of 1:1 L-glycine/D-xylose 10^{-3} M H_2O/CH_3OH 3:1 solution; b) 1 mL of 1:1 L-glycine/D-glucose 10^{-3} M H_2O/CH_3OH 3:1 solution; c) 1 mL of 1:1 L-glycine/D-fructose 10^{-3} M H_2O/CH_3OH 3:1 solution.

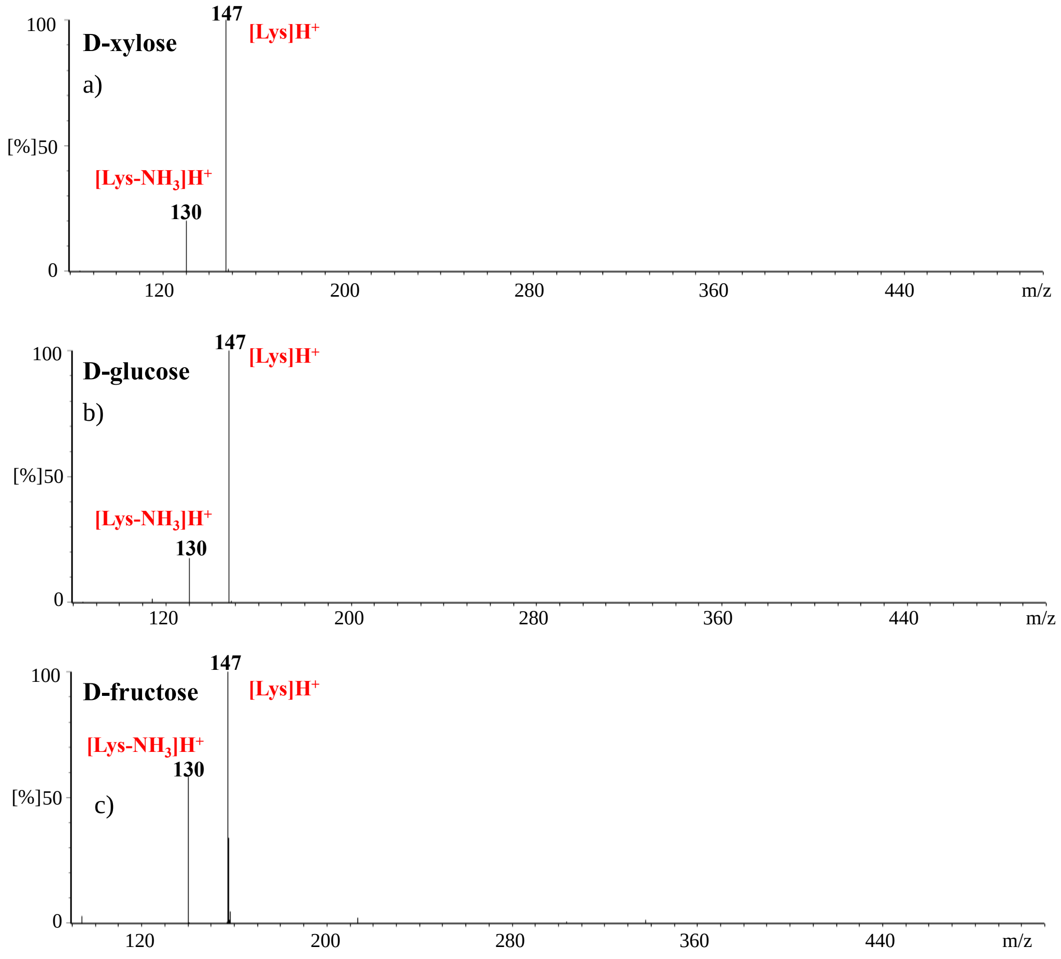

Figure S2. ESI mass spectra of the starting solutions: a) 1 mL of 1:1 L-lysine/D-xylose 10^{-3} M H_2O/CH_3OH 3:1 solution b) 1 mL of 1:1 L-lysine/D-glucose 10^{-3} M H_2O/CH_3OH 3:1 solution, c) 1 mL of 1:1 L-lysine/D-fructose 10^{-3} M H_2O/CH_3OH 3:1 solution.

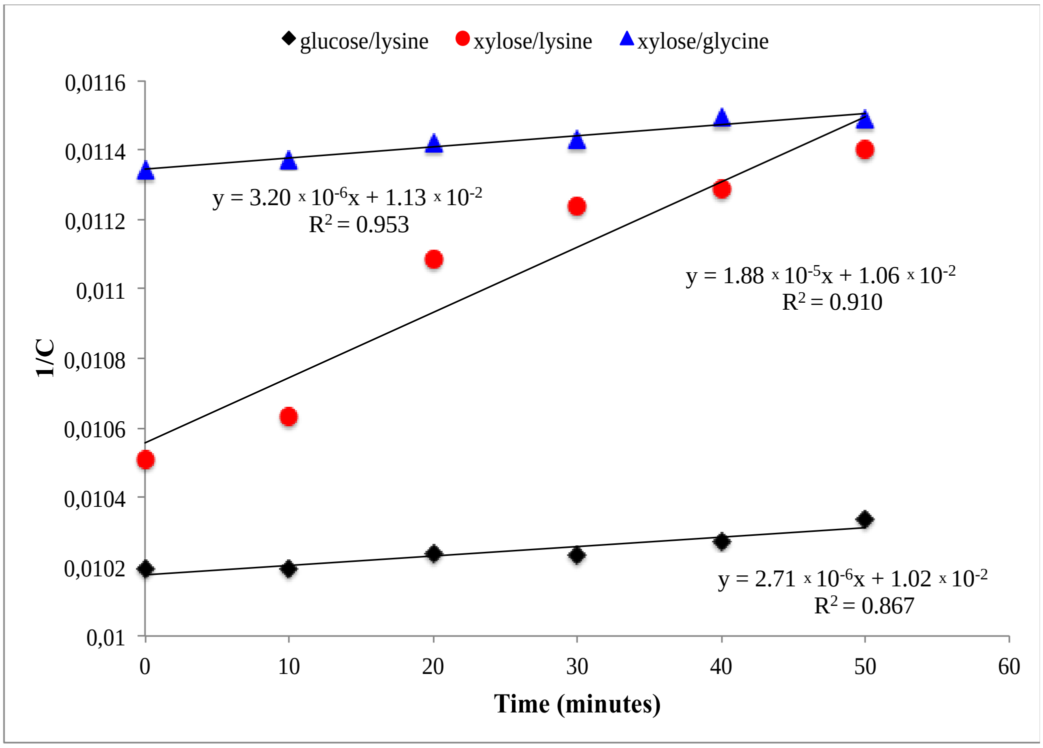

Figure S3. Kinetic plots of the reactions performed in bulk.
